# Timing of Neonatal Discharge and Unplanned Readmission to PICUs Among Infants Born Preterm

**DOI:** 10.1001/jamanetworkopen.2024.44909

**Published:** 2024-11-14

**Authors:** Tim J. van Hasselt, Yuhe Wang, Chris Gale, Shalini Ojha, Cheryl Battersby, Peter Davis, Hari Krishnan Kanthimathinathan, Elizabeth S. Draper, Sarah E. Seaton, For the United Kingdom Neonatal Collaborative and the Paediatric Critical Care Society Study Group (PCCS-SG)

**Affiliations:** 1 Department of Population Health Sciences, University of Leicester, Leicester, United Kingdom; 2 Neonatal Medicine, School of Public Health, Faculty of Medicine, Imperial College London, London, United Kingdom; 3 Centre for Paediatrics and Child Health, Imperial College London, London, United Kingdom; 4 Faculty of Medicine & Health Sciences, University of Nottingham, Nottingham, United Kingdom; 5 Paediatric Intensive Care Unit, Bristol Royal Hospital for Children, University Hospitals Bristol and Weston NHS Foundation Trust, Bristol, United Kingdom; 6 Paediatric Intensive Care Unit, Birmingham Women’s and Children’s NHS Foundation Trust, Birmingham, United Kingdom

## Abstract

**Importance:**

Children born very preterm (<32 weeks) are at risk of ongoing morbidity and admission to pediatric intensive care units (PICUs) in childhood. However, the influence of the timing of neonatal discharge on unplanned PICU admission has not been established.

**Objective:**

To examine whether the timing of neonatal discharge (postmenstrual age and season) is associated with subsequent unplanned PICU admission.

**Design, Setting, and Participants:**

This retrospective cohort study used linked national data from the National Neonatal Research Database and Paediatric Intensive Care Audit Network (PICANet) for children born from January 2013 to December 2018 at 22 to 31 weeks’ gestational age who were admitted to a neonatal unit in England and Wales and were discharged home at 34 weeks’ postmenstrual age or later. All National Health Service (NHS) neonatal units and PICUs in England and Wales were included. Children were followed up until 2 years of chronological age. Data analysis was conducted from October 2023 to August 2024.

**Exposures:**

Timing of discharge.

**Main Outcomes and Measures:**

The primary outcome was unplanned PICU admission between neonatal discharge and chronological age 2 years to any PICU within England and Wales. Survival analysis using a flexible parametric model was conducted with season of discharge (time-dependent factor), gestation, sex, birth weight less than the 10th centile, bronchopulmonary dysplasia, necrotizing enterocolitis, brain injury, and earlier neonatal discharge (lower quartile of postmenstrual age at discharge for gestation) as variables.

**Results:**

Of 39 938 children discharged home (median [IQR] gestational age, 29 [27-31] weeks; 21 602 [54.1%] male), 1878 (4.7%) had unplanned PICU admission. More than half of admissions occurred within 50 days of neonatal discharge (1080 [57.5%]). Compared with summer, the risk of unplanned PICU admission following neonatal discharge was 2.58 times higher in winter and 2.35 times higher in autumn (winter: adjusted hazard ratio [aHR], 2.58; 95% CI, 1.68-3.95; autumn: aHR, 2.35; 95% CI, 1.84-2.99). Among children born at 28 to 31 weeks’ gestational age, earlier neonatal discharge was associated with increased risk (aHR, 1.30; 95% CI, 1.13-1.49), but this was not true for children born younger than 28 weeks’ gestational age.

**Conclusions and Relevance:**

In this retrospective cohort study of preterm children, autumn and winter discharge were associated with the highest risk of unplanned PICU admission following neonatal discharge. For children born at 28 to 31 weeks’ gestational age, discharge at lower postmenstrual age was also associated with increased risk. Further work is required to understand whether delaying neonatal discharge for some children born at 28 to 31 weeks’ gestational age is beneficial and to consider the wider costs and implications of prolonging neonatal care.

## Key Points

**Question:**

Among very preterm children, is the timing of neonatal discharge associated with subsequent unplanned pediatric intensive care unit (PICU) admission before the age of 2 years?

**Findings:**

In this cohort study with 39 938 children, there was twice the risk of unplanned PICU admission following neonatal discharge in autumn and winter compared with summer for children born younger than 32 weeks’ gestational age. Among children born between 28 and 31 weeks’ gestational age, discharge at a younger age was associated with a higher estimated risk of PICU admission.

**Meaning:**

Clinicians and families should be aware of the increased risk of subsequent intensive care admission among infants born younger than 32 weeks’ gestation following discharge from neonatal units during the autumn and winter seasons and explore mitigations where possible.

## Teaser

This cohort study examines whether the season and postmenstrual age of preterm infants discharged from neonatal units are associated with unplanned readmission to pediatric intensive care units (PICUs) in England and Wales.

## Introduction

Due to the ongoing effects of prematurity, children born very preterm (<32 weeks’ gestation) are at increased risk of unplanned admission to pediatric intensive care units (PICUs) after discharge home from neonatal care due to respiratory disease.^1,2^ The incidence of viral infections such as respiratory syncytial virus (RSV) shows marked seasonal variation, increasing over autumn and winter in northern hemisphere countries.^3,4^ However, there are no existing data on how the season of neonatal discharge affects the risk of PICU admission for very preterm children. Understanding this may influence discussions with parents in the lead up to neonatal discharge.

Generally, babies born at earlier gestational ages are discharged home at later postmenstrual ages (PMA, also known as corrected gestational age)^5^; however, considerable variation has been observed both within and between countries, with differences of up to 3 weeks in PMA at discharge for babies of similar gestational ages.^5,6,7,8^ Decision-making around discharge is based on factors including cardiorespiratory stability, thermoregulation, nutrition, and support in the community, variably assessed between neonatal units.^9^ To our knowledge, no study has evaluated whether earlier discharge of very preterm babies is associated with increased risk of PICU admission. We aimed to use linked national routinely collected data to investigate the association of season and PMA at neonatal discharge with unplanned PICU admissions up to the age of 2 years among children born younger than 32 weeks’ gestational age and discharged from neonatal care.

## Methods

We have previously described this birth cohort and data linkage.^2^ The National Neonatal Research Database (NNRD) provided data for all children born younger than 32 weeks’ gestational age from January 1, 2013, to December 31, 2018, in England and Wales. The Paediatric Intensive Care Audit Network (PICANet) provided data for all children admitted to PICUs in England and Wales before the chronological age of 2 years. NHS Digital (now NHS England) performed data linkage using personal identifiers (NHS number present in >99% of children, date of birth, surname, postcode) and provided pseudonymized linked data, allowing identification of children discharged home from neonatal units and subsequent unplanned (ie, nonelective) PICU admissions from home 24 hours or later after neonatal discharge. Planned or unplanned status is entered by admitting clinicians following PICANet definitions.^10^

Ethical approval for this study was provided by the East of England committee and the Confidentiality Advisory Group. Informed consent was not required for this study because it used secondary data. This study is reported following the Strengthening the Reporting of Observational Studies in Epidemiology (STROBE) reporting guideline.

The NNRD captures demographic and clinical data for all neonatal unit admissions, with complete coverage for England and Wales from 2013.^11^ PICANet is a national audit database that has collected demographic and clinical data for all PICU admissions in England and Wales since 2003.^10^ Both datasets undergo verification and data cleaning.^10,11^

### Included Population for Analysis

We included children born at 22 to 31 weeks’ completed gestation who were admitted for neonatal care on day 1 of life and survived to discharge home from neonatal care. We excluded babies whose neonatal discharge home occurred before 34 weeks’ PMA, as maturation milestones, such as unaided temperature regulation and oral feeding, generally take place after this point.^6,12,13,14^ Therefore, discharge before 34 weeks’ PMA may reflect erroneous data or unusual discharge practices, and we intended for our category of earlier neonatal discharge to reflect variation in usual practice within a UK context.

Birthweights more than 3 SDs from the median for gestation and sex were replaced with missing values but were not excluded.^15^ Small for gestational age (SGA) was defined as birth weight lower than the 10th centile for gestation and sex using existing thresholds.^16,17^ Values for weight at neonatal discharge were replaced with missing if they were less than 1000 g or greater than 5000 g or more than 3 SDs from the median.

### Statistical Analysis

We performed summary statistics for the cohort, presenting frequencies and percentages for categorical variables, means and SDs for normally distributed variables, and medians and IQRs for nonnormally distributed variables. We compared the characteristics of children with unplanned PICU admissions with those without, using χ^2^ tests for categorical variables, *t* tests for normally distributed variables, and the Wilcoxon rank test for nonnormally distributed variables.

We undertook survival analysis using the outcome of first unplanned PICU admission from the point of neonatal discharge until age 2 years (chronological), based on a complete case analysis. As we lacked data for children who moved outside the study region and for the timings of deaths outside the PICU, we did not censor for these events. We used a flexible parametric model,^18^ adjusting for variables as identified by our clinical advisory group (eTable 1 in Supplement 1). Season of neonatal discharge was included as a categorical variable (spring, March to May; summer, June to August; autumn or fall, September to November; and winter, December to February). Per a prespecified definition developed from previous work,^5^ earlier neonatal discharge included babies discharged at the lowest quartile of PMA at the point of neonatal discharge for their gestational age at birth, using observed data from this cohort after the previously described exclusions. Other planned variables included gestational age at birth in completed weeks (as a categorical variable), sex, SGA, bronchopulmonary dysplasia (BPD; respiratory support or oxygen use at 36 weeks PMA), severe necrotizing enterocolitis (NEC) requiring surgery,^19^ and neonatal brain injury (grade III or IV intraventricular hemorrhage, cystic periventricular leukomalacia, hydrocephalus, or meningitis^20^).

We tested the proportional hazard assumption and identified time-dependent associations in which the relationship between a variable and the hazard ratio (HR) varies over the observed time course, using Schoenfeld residual test and plots.^21^ Generally, any *P* value less than .05 in the test suggests a time-dependent effect. However, due to the large sample size, *P* values may be misleading, so the final decision was based on visual assessment of the Schoenfeld residual plots for deviation. Due to the use of cubic restricted splines, degrees of freedom were chosen based on Akaike information criteria and bayesian information criteria. We calculated E-values for HRs using methods previously published.^22^ The resulting E-values represent the minimum magnitude of an HR from an unmeasured confounder required to provide an alternative explanation for the observed effect.

We performed subgroup analysis for babies born younger than 28 weeks’ gestational age and those born at 28 to 31 completed weeks’ gestational age, as previous work suggests that unplanned PICU admission may occur earlier for those born later.^2^ We also performed sensitivity analyses using late neonatal discharge (ie, discharge occurring >75th centile of PMA for neonatal discharges at each gestation) and including babies discharged at 33 weeks’ PMA or later.

We set the level of statistical significance at *P* < .05, and tests for significance were 2-tailed. All statistical analyses were conducted using Stata version 18.0 (StataCorp).

## Results

**Figure 1.**
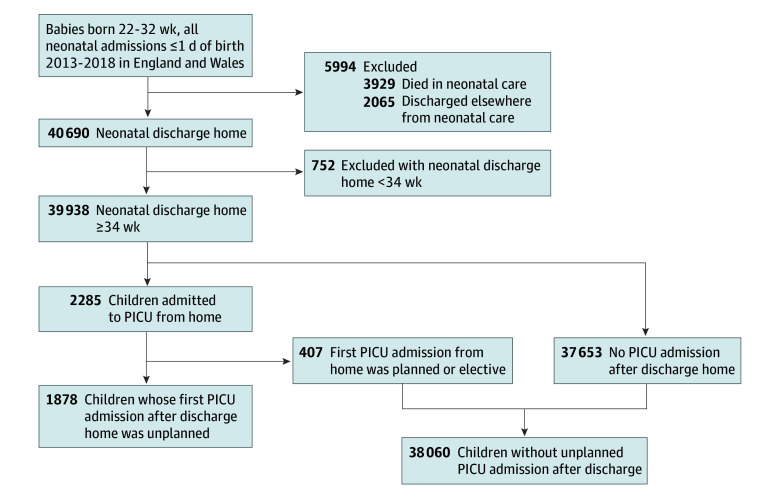
Study Flowchart PICU indicates pediatric intensive care unit.

From the 46 684 children born at 22 to 31 weeks’ gestational age between 2013 and 2018 and admitted to neonatal units within England and Wales within the first day of life, we excluded 3939 children who died in neonatal care, 2065 children discharged to other health care settings, and 752 children discharged home earlier than 34 weeks’ PMA, leaving a cohort of 39 938 children discharged home from neonatal care (median [IQR] gestational age, 29 [27-31] weeks; 21 602 [54.1%] male) (Figure 1). Of these children, 2285 (5.7%) were admitted to PICU after discharge home and before the age of 2 years, of whom 1878 had first admissions that were unplanned (4.7% of the cohort and 82.2% of children with PICU admissions). Unplanned admissions were most commonly due to respiratory disease (any respiratory disease, 1310 [69.8%]; bronchiolitis, 995 [53.0%]); other causes included other infections (212 [11.3%]) and cardiovascular disease (81 [4.3%]) (eTable 2 in Supplement 1). During the first unplanned PICU admission, most children received invasive ventilation (1422 [75.7%]), the median (IQR) length of stay was 6 (4-9) days, and 54 children (2.9%) died in the PICU. Of survivors, 425 children (23.3%) had at least 1 PICU readmission of any type before age 2 years. These outcomes are presented by gestational age subgroup (<28 weeks and 28-31 weeks) and earlier discharge status in eTable 3 in Supplement 1. There were 128 children (0.3%) who died before age 2 years who were not admitted to PICU.

Compared with children without unplanned PICU admission, among those with unplanned PICU admissions, a greater percentage were male (20 418 [53.8%] vs 1121 [59.7%]; *P* < .001), were born earlier (<24 weeks’ gestational age: 474 [1.2%] vs 54 [2.9%]; *P* < .001), and had lower mean (SD) birth weight (1246 [358] g vs 1147 [381] g; *P* < .001) (Table 1). The PICU group, compared with the non-PICU group, also had more neonatal morbidity, such as BPD (810 [43.1%] vs 10 799 [28.4%]; *P* < .001). The overall cohort had similar numbers of neonatal discharges throughout the year; however, among children subsequently admitted to PICU there were greater percentages discharged in autumn (602 [32.1%]) and winter (533 [28.4%]) compared with summer (346 [18.4%]) (*P* < .001). A similar proportion of children were discharged earlier, ie, less than the 25th centile PMA at neonatal discharge for their gestation (children with unplanned PICU admission, 485 [25.8%]; children without unplanned PICU admission, 9950 [26.1%]), and mean (SD) weight at neonatal discharge was slightly greater in children with admissions (2439 [529] g) compared with those without (2350 [491] g) (*P* < .001).

**Table 1.** Birth and Neonatal Characteristics of the Cohort^a^

Characteristic	Children, No. (%) (N = 39 938)		*P* value
	No unplanned PICU admission after discharge home	Unplanned PICU admission after discharge home	
Total	38 060 (95.3)	1878 (4.7)	NA
Sex			
Male	20 481 (53.8)	1121 (59.7)	<.001
Female	17 559 (46.1)	755 (40.2)	
Missing	20 (0.1)	2 (0.1)	NA
Gestation, wk			
<24	474 (1.2)	54 (2.9)	<.001
24	1278 (3.4)	137 (7.3)	
25	1862 (4.9)	165 (8.8)	
26	2584 (6.8)	195 (10.4)	
27	3514 (9.2)	184 (9.8)	
28	4804 (12.6)	229 (12.2)	
29	5756 (15.1)	258 (13.7)	
30	7617 (20.0)	308 (16.4)	
31	10 171 (26.7)	348 (18.5)	
Birth weight, g			
Mean (SD)	1246 (358.0)	1147 (381.4)	<.001
Small for gestational age (<10th centile)	3061 (8.0)	170 (9.1)	.12
Missing	151 (0.4)	8 (0.4)	NA
Weight at neonatal discharge, g			
Mean (SD)	2350.0 (490.5)	2438.9 (529.4)	<.001
Missing	1068 (2.8)	104 (5.5)	NA
Multiple birth			
Singleton	28 015 (73.6)	1411 (75.1)	.14
Multiple	10 045 (26.4)	467 (24.9)	
Antenatal steroids			
None	1920 (5.0)	109 (5.8)	.32
Complete	26 918 (70.7)	1310 (69.8)	
Incomplete	6916 (18.2)	337 (17.9)	
Missing	2306 (6.1)	122 (6.5)	NA
Mode of delivery			
Vaginal delivery	13 273 (34.9)	727 (38.7)	<.001
Instrumental vaginal delivery	1013 (2.7)	34 (1.8)	
Cesarean delivery	22 209 (58.4)	1028 (54.7)	
Missing	1565 (4.1)	89 (4.7)	NA
BPD^b^			
None	27 072 (71.1)	1052 (56.0)	<.001
Present	10 799 (28.4)	810 (43.1)	
Missing	189 (0.5)	16 (0.9)	NA
Severe NEC^c^			
Present	591 (1.6)	64 (3.4)	<.001
Brain injury			
Present	2217 (5.8)	206 (11.0)	<.001
Season of neonatal discharge			
Spring (March to May)	9506 (25.0)	397 (21.1)	<.001
Summer (June to August)	9991 (26.3)	346 (18.4)	
Autumn (September to November)	9210 (24.2)	602 (32.1)	
Winter (December to February)	9353 (24.6)	533 (28.4)	
Neonatal discharge timing			
Earlier (<25th centile PMA)	9950 (26.1)	485 (25.8)	<.001
Expected (≥25th to <75th centile PMA)	18 520 (48.7)	835 (44.5)	
Late (≥75th centile PMA)	9590 (25.2)	558 (29.7)	

Abbreviations: BPD, bronchopulmonary dysplasia; NA, not applicable; NEC, necrotizing enterocolitis; PICU, pediatric intensive care unit; PMA, postmenstrual age.

^a^ The cohort consisted of children born earlier than 32 weeks’ gestational age between January 2013 and December 2018 in England and Wales who were discharged home from neonatal care at 34 weeks’ PMA or later.

^b^ Category includes children with BPD requiring oxygen or respiratory support at 36 weeks’ PMA.

^c^ Severe NEC indicates NEC requiring surgery.

We used a flexible parametric model for survival analysis of unplanned PICU admission, adjusting for gestation, sex, SGA, BPD, severe NEC, brain injury, season of neonatal discharge, and earlier discharge. After exclusion for missing data, which was minimal (<1% across variables) (Table 1), there were 39 556 children (99.0%) included in complete case analysis. Based on visual inspection of the Schoenfeld residual plots (eFigure 1 in Supplement 1), season of discharge was modeled as a time-dependent variable. We used 3 degrees of freedom for the baseline hazard, and 2 for the time-dependent term. Therefore, as HRs varied during the follow-up period, we report the HR for season at day 1 following neonatal discharge.

Lower gestational age at birth was associated with increased hazard of unplanned PICU admission: the adjusted HR (aHR) was 2.10 (95% CI, 1.54-2.88) for children born before 24 weeks’ gestation compared with those born at 31 weeks’ gestation (Table 2). Presence of major neonatal morbidity, such as BPD, was associated with increased risk of unplanned PICU admission (BPD: aHR, 1.44; 95% CI, 1.27-1.63), with similar increases for severe NEC (aHR, 1.44; 95% CI, 1.11-1.87) and brain injury (aHR, 1.38; 95% CI, 1.19-1.61). Similarly, the presence of SGA was associated with an increased risk (aHR, 1.21; 95% CI, 1.03-1.42). Earlier neonatal discharge was also associated with increased risk (aHR, 1.19; 95% CI, 1.06-1.33). eTable 4 in Supplement 1 presents thresholds for earlier discharge by gestation. Compared with neonatal discharge in summer, the risks were twice as great for discharge in autumn (aHR, 2.35; 95% CI, 1.84-2.99) and winter (aHR, 2.58; 95% CI, 1.68-3.95). Examining these HRs and their E-values, greater magnitude unmeasured confounders would be required to explain the increase in hazard of unplanned PICU admission and autumn discharge (E-value, 4.13), BPD (E-value, 2.23), or birth earlier than 24 weeks’ gestational age (E-value, 3.63) compared with that required to explain early neonatal discharge (E-value, 1.66).

**Table 2.** Flexible Parametric Model for Unplanned PICU Admission From Home Among 39 556 Children^a^

Variable	Adjusted hazard ratio (95% CI)	*P* value	E-value
Gestation at birth, wk			
<24	2.10 (1.54-2.88)	<.001	3.63
24	2.13 (1.70-2.67)	<.001	3.68
25	1.82 (1.48-2.25)	<.001	3.05
26	1.70 (1.40-2.06)	<.001	2.79
27	1.27 (1.05-1.53)	.01	1.85
28	1.24 (1.05-1.48)	.01	1.79
29	1.23 (1.05-1.45)	.01	1.77
30	1.15 (0.99-1.35)	.07	1.57
31	1 [Reference]	NA	NA
Sex			
Male	1.25 (1.14-1.37)	<.001	1.81
Female	1 [Reference]	NA	NA
Small for gestational age			
Present	1.21 (1.03-1.42)	.02	1.72
BPD^b^			
Present	1.44(1.27-1.63)	<.001	2.23
Severe NEC^c^			
Present	1.44 (1.11-1.87)	.006	2.24
Brain injury			
Present	1.38 (1.19-1.61)	<.001	2.11
Neonatal discharge timing			
Earlier (<25th centile PMA)	1.19 (1.06-1.33)	.003	1.66
Not early	1 [Reference]	NA	NA
Season of neonatal discharge^d^			
Spring	0.96 (0.75-1.24)	NA	1.25
Summer	1 [Reference]	NA	NA
Autumn	2.35 (1.84-2.99)	NA	4.13
Winter	2.58 (1.68-3.95)	NA	4.6

Abbreviations: BPD, bronchopulmonary dysplasia; NEC, necrotizing enterocolitis; PICU, pediatric intensive care unit; PMA, postmenstrual age.

^a^ Only children with complete data were included in this model.

^b^ Category includes children with BPD requiring oxygen or respiratory support at 36 weeks’ PMA.

^c^ Severe NEC indicates NEC requiring surgery.

^d^ Hazard ratio for season shown as estimated hazard ratio at day 1 due to modeling for time-dependent effect.

**Figure 2.**
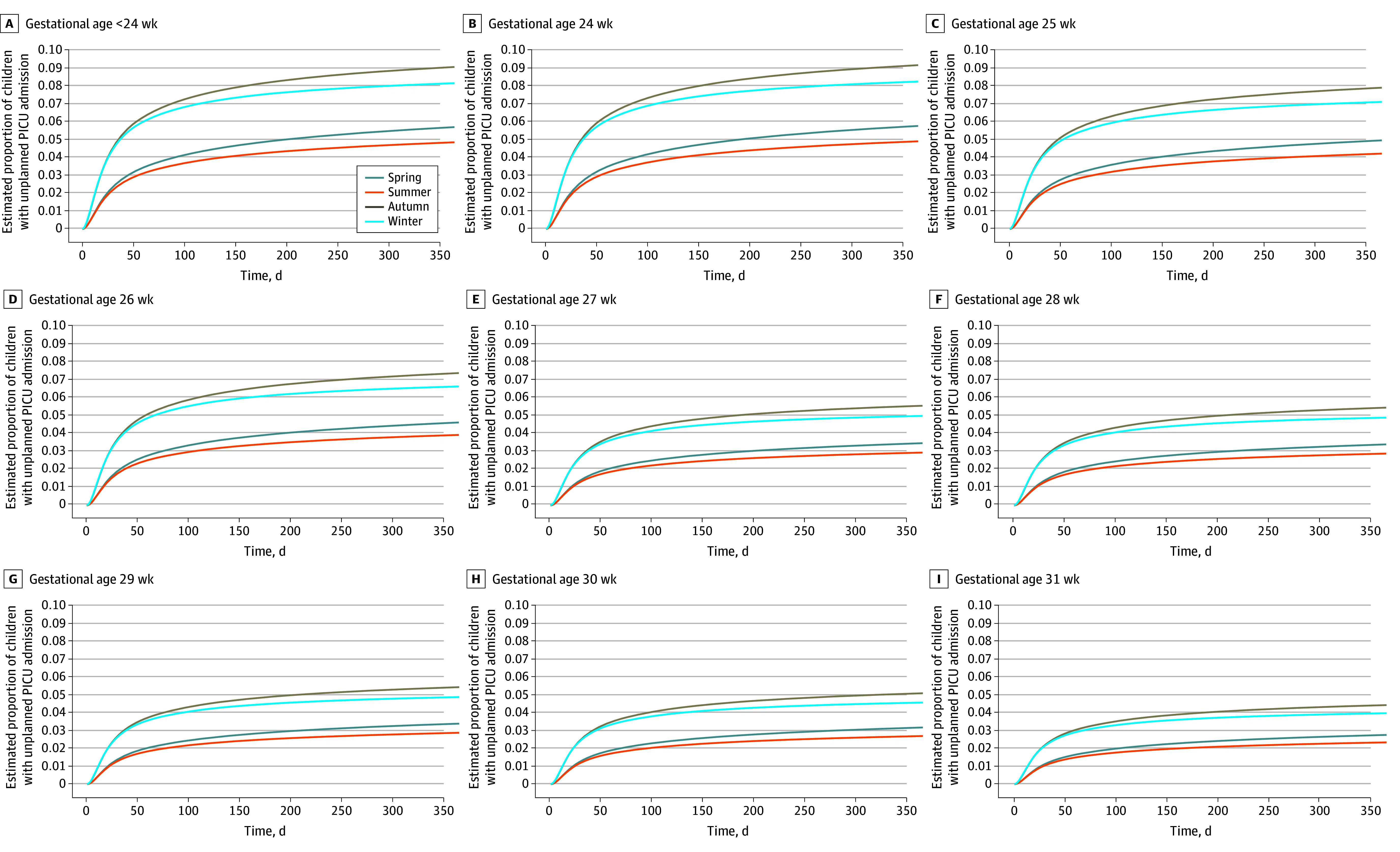
Flexible Parametric Model Estimates for Unplanned Pediatric Intensive Care Unit (PICU) Admission From Home in the First Year Following Neonatal Discharge, by Gestation at Birth in Completed Weeks and Season of Neonatal Discharge

Outputs from this model were used to estimate percentages of children with unplanned admission to PICU before age 2 years by gestational age at birth and by season of neonatal discharge (Table 3). For children born earlier than 24 weeks’ gestation, 5.4% (95% CI, 4.0%-7.2%) were estimated to have PICU admission following summer discharge from neonatal care, increasing to 8.6% (95% CI, 6.5%-11.3%) for those discharged in winter and 9.8% (95% CI, 7.4%-12.9%) in autumn. For children born at 31 weeks’ gestational age, 2.6% (95% CI, 2.2%-3.0%) were estimated to be admitted to PICU if discharged in summer, increasing to 4.2% (95% CI, 3.7%-4.8%) for winter discharge and 4.8% (95% CI, 4.2%-5.4%) for autumn discharge. Despite the HR at day 1 following discharge being greatest for winter discharge, autumn discharge had the greatest estimated cumulative risk for the whole time period due to time-dependent associations (eFigure 2 in Supplement 1). We also presented the estimated cumulative probabilities graphically (Figure 2). Unplanned PICU admissions tended to occur early within the follow-up time, with more than half of admissions occurring within the first 50 days (1080 of 1878 [57.5%]), across all gestations and seasons; therefore, we showed only the first year of follow-up, as PICU admission was less common after this point.

**Table 3.** Estimated Percentage of Children With Unplanned PICU Admission From Home Before Age 2 Years, by Gestational Age at Birth and Season of Neonatal Discharge

Gestation at birth, wk	Estimated % with unplanned PICU admission (95% CI), by season of neonatal discharge			
	Spring	Summer	Autumn	Winter
<24	6.5 (4.8-8.6)	5.4 (4.0-7.2)	9.8 (7.4-12.9)	8.6 (6.5-11.3)
24	6.5 (5.4-7.9)	5.4 (4.4-6.6)	9.9 (8.2-11.9)	8.7 (7.2-10.5)
25	5.6 (4.7-6.7)	4.7 (3.9-5.6)	8.5 (7.2-10.1)	7.5 (6.3-8.9)
26	5.3 (4.5-6.2)	4.4 (3.7-5.2)	8.0 (6.8-9.3)	7.0 (6.0-8.2)
27	3.9 (3.3-4.7)	3.3 (2.8-3.9)	6.0 (5.1-7.0)	5.3 (4.5-6.2)
28	3.9 (3.3-4.5)	3.2 (2.7-3.8)	5.9 (5.1-6.8)	5.2 (4.5-6.0)
29	3.8 (3.3-4.5)	3.2 (2.7-3.7)	5.9 (5.1-6.7)	5.1 (4.5-5.9)
30	3.6 (3.1-4.1)	3.0 (2.6-3.5)	5.5 (4.8-6.2)	4.8 (4.2-5.5)
31	3.1 (2.7-3.6)	2.6 (2.2-3.0)	4.8 (4.2-5.4)	4.2 (3.7-4.8)

Abbreviation: PICU, pediatric intensive care unit.

While the directions of other associations were consistently observed across primary and subgroup analyses, the increase in HR for children with earlier neonatal discharge was observed in the subgroup of children born 28 to 31 weeks’ gestation (aHR, 1.30; 95% CI, 1.13-1.49) but not for those born earlier than 28 weeks’ gestation (aHR, 1.01; 95% CI, 0.83-1.23) (eTable 5 in Supplement 1). For children born at 28 weeks’ gestation, earlier discharge was defined as occurring before 36.1 weeks’ PMA; for those born at 31 weeks’ gestation, it was defined as occurring before 35.3 weeks’ PMA. From subgroup analysis, the estimated probability of unplanned PICU admission for a child born at 28 weeks’ gestation and going home in autumn without earlier neonatal discharge was 5.7%, this increased to 7.3% if that child was discharged earlier from neonatal care. For children born at 31 weeks’ gestation and discharged in autumn, the estimated probability was 4.6% for those without earlier discharge, and 5.9% for those discharged earlier.

In a further separate analysis, late neonatal discharge (>75th centile PMA) was associated with increased risk for unplanned PICU admission compared with discharge at 25th centile or greater to less than 75th centile PMA for children born earlier than 28 weeks’ gestation (aHR, 1.24; 95% CI, 1.04-1.47) but not for children born at 28 to 31 weeks’ gestation (aHR, 0.98; 95% CI, 0.82-1.15) (eTables 6 and 7 in Supplement 1). The findings for earlier discharge (<25th centile PMA) were similar to the primary analysis.

In a sensitivity analysis using month of neonatal discharge rather than season, the resulting aHRs were similar, and using July as the reference, the greatest risk following neonatal discharge was observed for November (aHR, 2.40; 95% CI, 1.74-3.32) (eFigure 3 in Supplement 1). Finally, we repeated analysis including neonatal discharges home at 33 weeks’ PMA or later (an additional 482 children); however, aHRs were unchanged (eTables 8 and 9 in Supplement 1).

## Discussion

In this study, we used a survival analysis approach to model the hazard of unplanned PICU admission after neonatal discharge for very preterm children and observed a clear association between the timing of neonatal discharge and PICU admission, after adjustment for gestation, sex, SGA, and neonatal morbidities. To our knowledge, this is the first time the variation in risk of unplanned PICU admission depending on the season of neonatal discharge has been quantified. In addition, we identified a novel association between this risk and earlier neonatal discharge in babies born at 28 to 31 weeks’ gestation, although this was not present among babies born earlier than 28 weeks’ gestation, who by contrast were noted to have increased risk with later neonatal discharge.

The association between lower gestational age at birth and rehospitalization after neonatal discharge has previously been demonstrated.^23,24^ A previous study of 512 babies born weighing less than 1250 g who received invasive mechanical ventilation after birth^25^ found that 58% were readmitted to hospital after discharge and 19% had PICU admission, although this was from the 2000s and less generalizable to the current very preterm population. Among a mixed population of preterm and full-term babies admitted to neonatal units, the lowest quartile of neonatal length of stay was associated with early hospital readmission (within 7 days)^26^; however, this was driven by the larger number of full-term babies. Unlike previous studies, we examined a contemporary national population of very preterm children, focused on PICU admissions, and used a novel application of flexible parametric analysis to quantify seasonal variation.

### Earlier Neonatal Discharge

We previously noted in this cohort that unplanned PICU admissions generally take place approximately 8 weeks after neonatal discharge for babies born at approximately 25 weeks’ gestation. However, for babies born at 31 weeks’ gestation, unplanned PICU admission occurred only a few weeks after neonatal discharge, on average before their due date.^2^ It may be that these relatively less-preterm babies appear to be ready for neonatal discharge at around 34 to 35 weeks’ PMA but are still more vulnerable to respiratory tract infections than they would be 1 or 2 weeks later. There is variation in PMA at neonatal discharge both nationally and internationally,^5,6,7,8^ eg, children born at 28 weeks’ gestation are commonly discharged between 36 and 40 weeks’ PMA,^8^ and there is a lack of evidence for practice.

We found that babies born earlier than 28 weeks’ gestation had increased risk of PICU admission with later neonatal discharge, likely due to medical complexity necessitating prolonged stays. This may not be completely captured by adjusting for a limited number of key neonatal morbidities.

### Seasonal Findings

Consistent with the well-established seasonality of pediatric respiratory tract infections and PICU admissions,^4,27^ we observed an increased hazard of unplanned PICU admission among very preterm born children discharged from neonatal care in autumn or winter. The greatest impact on cumulative risk was due to the season at the time of neonatal discharge and the season that follows. Children discharged in autumn continue to be exposed to respiratory viruses into winter, while those discharged in winter have a reducing risk of exposure as the season changes to spring.

This novel data regarding the estimated risk of PICU admission may be helpful to clinicians and families, particularly during autumn and winter. Using data to identify and explain this risk could form part of standard neonatal discharge planning practices.

### Policy Implications

While the observed mortality rate in PICU in our cohort was low (2.9%, compared with 3.4%, the overall UK mortality rate for PICU admissions in 2018), as expected for mainly respiratory admissions, the median length of stay (6 days) was longer (2-3 days for the overall PICU population in 2018).^10^ Given that admissions are more likely during times of high PICU bed occupancy (autumn and winter),^10^ these unplanned admissions may result in rescheduling of elective surgery and transfers due to capacity.

It may be prudent to consider the individual risks and benefits of discharge prior to 35 weeks’ PMA during autumn and winter for babies born at 28 to 31 weeks’ gestation, given the observed small but significant association with unplanned PICU admission. Further prospective studies could explore whether this association is replicated and investigate the ideal timing for neonatal discharge, considering the wider clinical, societal, and economic implications of prolonging neonatal care.

One important consideration is that we did not have data on receipt of RSV prophylaxis. While other high-income countries have adopted universal use of the newer single-dose monoclonal antibody nirsevimab,^28,29^ the UK is planning maternal RSV immunization from 28 weeks.^30^ In our study, among children born at 28 to 31 weeks’ gestation, it is unlikely those who go home earlier would meet the criteria for palivizumab RSV prophylaxis, as the current UK guidelines still recommend that it is given to preterm babies who require supplemental oxygen or respiratory support at 36 weeks’ PMA.^31^ However, it is possible that higher-risk children eligible for palivizumab were protected from RSV and avoided or delayed PICU admission, and this may have been the case for children born earlier than 28 weeks’ gestation with earlier neonatal discharge. Moreover, behavioral changes in families of extremely preterm children may have reduced their exposure to respiratory viruses, although the evidence for the effectiveness of behavior modification to avoid viral infection in babies is unclear.^32^ Further research is required to understand the criteria used to decide whether an infant is suitable for discharge depending on season and whether clearer advice or other interventions for families going home at higher-risk periods could reduce respiratory infections. This could include additional follow-up and outreach support at home. As placental antibody transfer takes place in the third trimester,^33^ the clinical efficacy of maternal RSV vaccination among children born at 28 to 31 weeks’ gestation should be investigated, alongside the evaluation of alternative approaches, eg, administration of nirsevimab prior to discharge for all very preterm babies or those with incomplete maternal immunization.

### Limitations

This study has limitations. The use of national linked datasets allowed us to produce results that are generalizable to the neonatal and pediatric services of comparable high-income countries, based on detailed data from neonatal care. While both the NNRD and PICANet datasets undergo validation processes, all routinely collected data may be subject to missing data and misclassification, which if differential may lead to bias. We used variables with low levels of missing data (<5%).

While it is not possible to quantify missing data linkages, the presence of NHS numbers in more than 99% of children provided confidence in matching across datasets. There may have been children who moved outside of the study region (England and Wales) during the follow-up period and had PICU admissions that were not captured.

The NNRD does not include data on reasons for discharge decisions, and these may include both clinical and nonclinical reasons, such as family support, housing, or geographical constraints. Unmeasured confounders associated with respiratory tract infection not captured in these datasets include RSV prophylaxis, smoking within the household, presence of siblings, and attendance at childcare or nursery settings.^34,35,36^ We anticipate that future studies will make use of linkage of other datasets to investigate these factors and allow for the creation of predictive models effective at the individual level.

## Conclusions

In this cohort study, very preterm children had more than twice the risk of critical illness and requiring PICU admission following neonatal discharge in autumn and winter compared with summer—families should be aware of this risk. In addition, we found that discharge at earlier postmenstrual ages was associated with greater risk of unplanned PICU admissions for babies born at 28 to 31 weeks’ gestational age. More research is needed to further confirm these findings and explore potential explanations and mitigation strategies.

## Notes

Supplement 1. **eTable 1.** Members of Multidisciplinary Advisory Panel for Study Project **eTable 2.** Top 20 Most Frequent Primary Admission Diagnosis Codes for Unplanned PICU Admissions From Home **eTable 3.** Outcomes Within PICU by Gestation Group and Early Discharge Status, of 1878 Children With Unplanned PICU Admission After Neonatal Discharge **eTable 4.** Median and IQR PMAs at Neonatal Discharge **eTable 5.** Flexible Parametric Model for Unplanned PICU Admission From Home, Analysis for Children Born Earlier Than 24 Weeks’ to 27 Weeks’ Gestation and Those Born 28 to 31 Weeks’ Gestation **eTable 6.** Flexible Parametric Model for Unplanned PICU Admission From Home, Using Variables for Earlier and Late Neonatal Discharge (n = 39 556) **eTable 7.** Flexible Parametric Model for Unplanned PICU Admission From Home, Using Variables for Earlier and Late Neonatal Discharge, Analysis for Children Born Earlier Than 24 Weeks’ to 27 Weeks’ Gestation and Those Born 28 to 31 Weeks’ Gestation **eTable 8.** Flexible Parametric Model for Unplanned PICU Admission From Home, Including 40 038 Children Discharged Home at 33 weeks’ PMA or Later **eTable 9.** Flexible Parametric Model for Unplanned PICU Admission From Home, Including Children Discharged Home at 33 weeks’ PMA or Later, Analysis for Children Born Earlier Than 24 Weeks’ to 27 Weeks’ Gestation and Those Born 28 to 31 Weeks’ Gestation **eFigure 1.** Schoenfeld Plots **eFigure 2.** Hazard Ratio for Unplanned PICU Admission Over the First 100 Days From Neonatal Discharge for Season of Neonatal Discharge for Primary Analysis **eFigure 3.** Graph of Estimated Hazard Ratio for Unplanned PICU Admission on Day 1 Following Neonatal Discharge, by Month of Neonatal Discharge

Supplement 2.
Nonauthor Collaborators

Supplement 3.
Data Sharing Statement
