# Supplementary material for: Timing of Neonatal Discharge and Unplanned Readmission to PICUs Among Infants Born Preterm
Source: JAMA Netw Open. 2024 Nov 14;7(11):e2444909. doi: 10.1001/jamanetworkopen.2024.44909 (PMC11565260; doi:10.1001/jamanetworkopen.2024.44909)
Supplement: Supplement 1. — eTable 1. Members of Multidisciplinary Advisory Panel for Study Project eTable 2. Top 20 Most Frequent Primary Admission Diagnosis Codes for Unplanned PICU Admissions From Home eTable 3. Outcomes Within PICU by Gestation Group and Early Discharge Status, of 1878 Children With Unplanned PICU Admission After Neonatal Discharge eTable 4. Median and IQR PMAs at Neonatal Discharge eTable 5. Flexible Parametric Model for Unplanned PICU Admission From Home, Analysis for Children Born Earlier Than 24 Weeks’ to 27 Weeks’ Gestation and Those Born 28 to 31 Weeks’ Gestation eTable 6. Flexible Parametric Model for Unplanned PICU Admission From Home, Using Variables for Earlier and Late Neonatal Discharge (n = 39 556) eTable 7. Flexible Parametric Model for Unplanned PICU Admission From Home, Using Variables for Earlier and Late Neonatal Discharge, Analysis for Children Born Earlier Than 24 Weeks’ to 27 Weeks’ Gestation and Those Born 28 to 31 Weeks’ Gestation eTable 8. Flexible Parametric Model for Unplanned PICU Admission From Home, Including 40 038 Children Discharged Home at 33 weeks’ PMA or Later eTable 9. Flexible Parametric Model for Unplanned PICU Admission From Home, Including Children Discharged Home at 33 weeks’ PMA or Later, Analysis for Children Born Earlier Than 24 Weeks’ to 27 Weeks’ Gestation and Those Born 28 to 31 Weeks’ Gestation eFigure 1. Schoenfeld Plots eFigure 2. Hazard Ratio for Unplanned PICU Admission Over the First 100 Days From Neonatal Discharge for Season of Neonatal Discharge for Primary Analysis eFigure 3. Graph of Estimated Hazard Ratio for Unplanned PICU Admission on Day 1 Following Neonatal Discharge, by Month of Neonatal Discharge [file jamanetwopen-e2444909-s001.pdf]

Supplemental Online Content

van Hasselt TJ, Wang Y, Gale C, et al; United Kingdom Neonatal Collaborative and the Paediatric Critical Care Society Study Group (PCCS-SG). Timing of neonatal discharge and unplanned readmission to PICUs among infants born preterm. *JAMA Netw Open.* 2024;7(11):e2444909. doi:10.1001/jamanetworkopen.2024.44909

eTable 1. Members of Multidisciplinary Advisory Panel for Study Project

eTable 2. Top 20 Most Frequent Primary Admission Diagnosis Codes for Unplanned PICU Admissions From Home

eTable 3. Outcomes Within PICU by Gestation Group and Early Discharge Status, of 1878 Children With Unplanned PICU Admission After Neonatal Discharge

eTable 4. Median and IQR PMAs at Neonatal Discharge

eTable 5. Flexible Parametric Model for Unplanned PICU Admission From Home, Analysis for Children Born Earlier Than 24 Weeks' to 27 Weeks' Gestation and Those Born 28 to 31 Weeks' Gestation

eTable 6. Flexible Parametric Model for Unplanned PICU Admission From Home, Using Variables for Earlier and Late Neonatal Discharge (n = 39 556)

eTable 7. Flexible Parametric Model for Unplanned PICU Admission From Home, Using Variables for Earlier and Late Neonatal Discharge, Analysis for Children Born Earlier Than 24 Weeks' to 27 Weeks' Gestation and Those Born 28 to 31 Weeks' Gestation

eTable 8. Flexible Parametric Model for Unplanned PICU Admission From Home, Including 40 038 Children Discharged Home at 33 weeks' PMA or Later

eTable 9. Flexible Parametric Model for Unplanned PICU Admission From Home, Including Children Discharged Home at 33 weeks' PMA or Later, Analysis for Children Born Earlier Than 24 Weeks' to 27 Weeks' Gestation and Those Born 28 to 31 Weeks' Gestation

eFigure 1. Schoenfeld Plots

eFigure 2. Hazard Ratio for Unplanned PICU Admission Over the First 100 Days From Neonatal Discharge for Season of Neonatal Discharge for Primary Analysis

eFigure 3. Graph of Estimated Hazard Ratio for Unplanned PICU Admission on Day 1 Following Neonatal Discharge, by Month of Neonatal Discharge

This supplemental material has been provided by the authors to give readers additional information about their work.

© 2024 van Hasselt TJ et al. *JAMA Network Open.*

eTable 1. Members of Multidisciplinary Advisory Panel for Study Project

Name	Academic/clinical role	Institution
Professor Jennifer J Kurinczuk	Professor Of Perinatal Epidemiology & Director	National Perinatal Epidemiology Unit (NPEU), University of Oxford
Dr Jonathan Cusack	Consultant Neonatologist	University Hospitals of Leicester
Dr Patrick Davies	Consultant in Paediatric Intensive Care, Honorary Assistant Professor of Paediatrics	Nottingham University Hospitals, University of Nottingham
Dr Cheryl Battersby	Clinical Senior Lecturer, Honorary Consultant Neonatologist	Neonatal Data Analysis Unit - Imperial College London, Chelsea and Westminster NHS Foundation Trust
Dr Peter Davis	Consultant Paediatric Intensivist	Bristol Royal Hospital for Children, University Hospitals Bristol and Weston NHS Foundation Trust
Professor Nicola Mackintosh	Professor in Social Science applied to Health	Department of Population Health Sciences, University of Leicester
Professor Joseph C Manning MBE	Professor of Nursing and Child Health, Charge Nurse, Paediatric Critical Care Outreach Team	School of Healthcare, University of Leicester, and Nottingham Children's Hospital
Professor Chris Gale	Professor of Neonatal Medicine, Honorary Consultant Neonatologist	Faculty of Medicine, School of Public Health, Imperial College London

© 2024 van Hasselt TJ et al. *JAMA Network Open.*

eTable 2. Top 20 Most Frequent Primary Admission Diagnosis Codes for Unplanned PICU Admissions From Home

Diagnosis (NHS Read Code)	n	%
Bronchiolitis	310	16.51
Acute bronchiolitis due to respiratory syncytial virus	241	12.83
Acute bronchiolitis	174	9.27
Respiratory insufficiency	72	3.83
Acute bronchiolitis due to other specified organisms	70	3.73
Sepsis	69	3.67
LRTI - Lower respiratory tract infection	61	3.25
Acute bronchiolitis NOS	49	2.61
Apnoea	37	1.97
Acute respiratory failure	34	1.81
Cardiac arrest	33	1.76
Acute viral bronchiolitis	30	1.6
Pneumonia	26	1.38
CI - Chest infection	25	1.33
Aspiration pneumonitis	18	0.96
Respiratory distress	18	0.96
SGS - Subglottic stenosis	18	0.96
Acute bronchitis or bronchiolitis NOS	17	0.91
Croup	16	0.85
Respiratory arrest	16	0.85

© 2024 van Hasselt TJ et al. *JAMA Network Open.*

eTable 3. Outcomes Within PICU by Gestation Group and Early Discharge Status, of 1878 Children With Unplanned PICU Admission After Neonatal Discharge

Gestation at birth	Earlier discharge status	n	Outcomes in PICU			
			Invasive mechanical ventilation on first unplanned PICU admission, n (%)	Median length of stay (interquartile range)	Mortality within PICU on first unplanned admission, n (%)*	Readmission to PICU before 2 years of age, n (%)
**<28 weeks**	**Earlier (<25th centile PMA)**	156	111 (71.2)	6 (4 to 10)	<5 (<3)	27 (17)
	**Not earlier**	579	421 (73.3)	7 (4 to 10)	25 (4)	173 (30)
**28-31 weeks**	**Earlier (<25th centile PMA)**	329	274 (83.3)	6 (4 to 9)	<5 (<3)	53 (16)
	**Not earlier**	814	616 (75.7)	6 (4 to 9)	20 (3)	172 (21)

***Results rounded for non-disclosure where cells have low frequencies**

© 2024 van Hasselt TJ et al. *JAMA Network Open.*

eTable 4. Median and IQR PMAs at Neonatal Discharge

Gestation at birth (weeks)	Median study PMA at neonatal discharge for cohort, weeks (IQR)	Threshold for earlier neonatal discharge, from the 25th centile of PMA at neonatal discharge by gestation	Median PMA at neonatal discharge for children with unplanned PICU admission, weeks (IQR)
**<24**	41.9 (40.1 to 44.7)	40.1	42.2 (40.7 to 45.1)
**24**	41.1 (39.1 to 43.6)	39.1	41.4 (39.2 to 44.8)
**25**	40.0 (38.3 to 42.1)	38.3	40.3 (38.6 to 42.6)
**26**	39.0 (37.4 to 41.0)	37.4	39.9 (38.0 to 42.3)
**27**	38.1 (36.7 to 40.1)	36.7	38.7 (36.6 to 41.0)
**28**	37.4 (36.1 to 39.3)	36.1	37.4 (35.9 to 40.3)
**29**	36.7 (35.7 to 38.3)	35.7	36.8 (35.9 to 39.4)
**30**	36.3 (35.4 to 37.6)	35.4	36.1 (35.4 to 37.4)
**31**	36.0 (35.3 to 37.0)	35.3	35.9 (35.1 to 37.0)

© 2024 van Hasselt TJ et al. *JAMA Network Open.*

eTable 5. Flexible Parametric Model for Unplanned PICU Admission From Home, Analysis for Children Born Earlier Than 24 Weeks' to 27 Weeks' Gestation and Those Born 28 to 31 Weeks' Gestation

		Subgroup: <24 to 27 weeks gestation (n=10,367)		Subgroup: 28 to 31 weeks gestation (n=29,198)	
**Variables**		**Adjusted hazard ratio (95% confidence interval)**	***p***	**Adjusted hazard ratio (95% confidence interval)**	***p***
**Gestation at birth (weeks)**	**<24**	1.72 (1.25 to 2.37)	0.001	-	-
	**24**	1.72 (1.36 to 2.17)	<0.001	-	-
	**25**	1.48 (1.19 to 1.84)	<0.001	-	-
	**26**	1.36 (1.11 to 1.66)	0.003	-	-
	**27**	Reference	-	-	-
	**28**	-	-	1.25 (1.05 to 1.49)	0.01
	**29**	-	-	1.24 (1.05 to 1.46)	0.01
	**30**	-	-	1.16 (0.99 to 1.35)	0.06
	**31**	-	-	Reference	-
**Sex**	**Male**	1.18 (1.02 to 1.37)	0.03	1.30 (1.15 to 1.47)	<0.001
	**Female**	Reference	-	Reference	-
**Small for gestational age**	**Present**	1.35 (0.99 to 1.83)	0.06	1.19 (0.98 to 1.44)	0.08
**BPD**	**Present**	1.49 (1.22 to 1.82)	<0.001	1.36 (1.16 to 1.60)	<0.001
**Severe NEC**	**Present**	1.33 (0.98 to 1.79)	0.07	1.86 (1.11 to 3.10)	0.02
**Brain injury**	**Present**	1.23 (1.01 to 1.48)	0.04	1.81 (1.40 to 2.33)	<0.001
**Neonatal discharge timing**	**Earlier (<25th centile PMA)**	1.01 (0.83 to 1.23)	0.89	1.30 (1.13 to 1.49)	<0.001
	**Not early**	Reference	-	Reference	-
		**Estimated hazard ratio on day 1**			
**Season of neonatal discharge (Estimated hazard ratio at day 1 from time-dependent effect model)***	**Spring**	1.04 (0.70 to 1.54)		0.91 (0.66 to 1.25)	
	**Summer**	Reference		Reference	
	**Autumn**	1.62 (1.09 to 2.41)		3.02 (2.22 to 4.11)	
	**Winter**	1.94 (0.97 to 3.91)		3.22 (1.88 to 5.52)	

BPD: bronchopulmonary dysplasia requiring oxygen at 36 weeks postmenstrual age
Severe NEC: necrotising enterocolitis requiring surgery
*Hazard ratio for season shown as estimated Hazard Ratio at Day 1 due to modelling for time-dependent effect

© 2024 van Hasselt TJ et al. *JAMA Network Open.*

eTable 6. Flexible Parametric Model for Unplanned PICU Admission From Home, Using Variables for Earlier and Late Neonatal Discharge (n = 39 556)

Variables		Adjusted hazard ratio (95% confidence interval)	*p*
**Gestation at birth (weeks)**	**<24**	2.16 (1.58 to 2.97)	<0.001
	**24**	2.19 (1.74 to 2.75)	<0.001
	**25**	1.85 (1.50 to 2.28)	<0.001
	**26**	1.73 (1.43 to 2.10)	<0.001
	**27**	1.29 (1.06 to 1.56)	0.009
	**28**	1.26 (1.06 to 1.50)	0.01
	**29**	1.24 (1.05 to 1.46)	0.01
	**30**	1.16 (0.99 to 1.35)	0.07
	**31**	Reference	-
**Sex**	**Male**	1.25 (1.14 to 1.37)	<0.001
	**Female**	Reference	-
**Small for gestational age**	**Present**	1.18 (1.00 to 1.39)	0.05
**BPD**	**Present**	1.40 (1.23 to 1.59)	<0.001
**Severe NEC**	**Present**	1.41 (1.08 to 1.83)	0.01
**Brain injury**	**Present**	1.37 (1.17 to 1.60)	<0.001
**Neonatal discharge timing**	**Earlier (<25th centile PMA)**	1.21 (1.08 to 1.36)	0.001
	**Expected (≥25th to <75th centile)**	Reference	-
	**Late (≥75th centile)**	1.08 (0.97 to 1.23)	0.15
		**Estimated hazard ratio on day 1**	
**Season of neonatal discharge (Estimated hazard ratio at day 1 from time-dependent effect model)***	**Spring**	0.96 (0.75 to 1.24)	
	**Summer**	Reference	
	**Autumn**	2.35 (1.84 to 2.99)	
	**Winter**	2.57 (1.68 to 3.95)	

BPD: bronchopulmonary dysplasia requiring oxygen at 36 weeks postmenstrual age
Severe NEC: necrotising enterocolitis requiring surgery
*Hazard ratio for season shown as estimated Hazard Ratio at Day 1 due to modelling for time-dependent effect

© 2024 van Hasselt TJ et al. *JAMA Network Open.*

eTable 7. Flexible Parametric Model for Unplanned PICU Admission From Home, Using Variables for Earlier and Late Neonatal Discharge, Analysis for Children Born Earlier Than 24 Weeks' to 27 Weeks' Gestation and Those Born 28 to 31 Weeks' Gestation

		Subgroup: <24 to 27 weeks gestation (n=10,367)		Subgroup: 28 to 31 weeks gestation (n=29,189)	
**Variables**		**Adjusted hazard ratio (95% confidence interval)**	***p***	**Adjusted hazard ratio (95% confidence interval)**	***p***
**Gestation at birth (weeks)**	**<24**	1.76 (1.28 to 2.42)	0.001	-	-
	**24**	1.75 (1.38 to 2.21)	<0.001	-	-
	**25**	1.46 (1.17 to 1.81)	0.001	-	-
	**26**	1.36 (1.11 to 1.67)	0.003	-	-
	**27**	Reference	-	-	-
	**28**	-	-	1.24 (1.04 to 1.49)	0.02
	**29**	-	-	1.24 (1.05 to 1.46)	0.01
	**30**	-	-	1.16 (0.99 to 1.35)	0.07
	**31**	-	-	Reference	-
**Sex**	**Male**	1.18 (1.02 to 1.37)	0.03	1.30 (1.15 to 1.47)	<0.001
	**Female**	Reference	-	Reference	-
**Small for gestational age**	**Present**	1.27 (0.93 to 1.73)	0.13	1.19 (0.98 to 1.46)	0.07
**BPD**	**Present**	1.43 (1.17 to 1.75)	<0.001	1.38 (1.16 to 1.64)	<0.001
**Severe NEC**	**Present**	1.25 (0.92 to 1.69)	0.16	1.87 (1.12 to 3.14)	0.02
**Brain injury**	**Present**	1.21 (1.00 to 1.46)	0.05	1.81 (1.41 to 2.34)	<0.001
**Neonatal discharge timing**	**Earlier (<25th centile PMA)**	1.09 (0.88 to 1.33)	0.43	1.29 (1.12 to 1.49)	<0.001
	**Expected (≥25th to <75th centile)**	Reference	-	Reference	-
	**Late (≥75th centile)**	1.24 (1.04 to 1.47)	0.02	0.98 (0.82 to 1.15)	0.76
		**Estimated hazard ratio on day 1**			
**Season of neonatal discharge (Estimated hazard ratio at day 1 from time-dependent effect model)***	**Spring**	1.03 (0.69 to 1.53)		0.91 (0.66 to 1.25)	
	**Summer**	Reference		Reference	
	**Autumn**	1.62 (1.09 to 2.41)		3.02 (2.22 to 4.11)	
	**Winter**	1.93 (0.96 to 3.88)		3.23 (1.88 to 5.52)	

BPD: bronchopulmonary dysplasia requiring oxygen at 36 weeks postmenstrual age
Severe NEC: necrotising enterocolitis requiring surgery
*Hazard ratio for season shown as estimated Hazard Ratio at Day 1 due to modelling for time-dependent effect

© 2024 van Hasselt TJ et al. *JAMA Network Open.*

eTable 8. Flexible Parametric Model for Unplanned PICU Admission From Home, Including 40 038 Children Discharged Home at 33 weeks' PMA or Later

Variables		Adjusted hazard ratio (95% confidence interval)	*p*
**Gestation at birth (weeks)**	**<24**	2.12 (1.55 to 2.91)	<0.001
	**24**	2.15 (1.71 to 2.70)	<0.001
	**25**	1.84 (1.49 to 2.27)	<0.001
	**26**	1.72 (1.42 to 2.09)	<0.001
	**27**	1.28 (1.06 to 1.55)	0.01
	**28**	1.25 (1.05 to 1.49)	0.01
	**29**	1.24 (1.06 to 1.46)	0.009
	**30**	1.15 (0.98 to 1.34)	0.08
	**31**	Reference	-
**Sex**	**Male**	1.26 (1.15 to 1.38)	<0.001
	**Female**	Reference	
**Small for gestational age**	**Present**	1.20 (1.02 to 1.41)	0.03
**BPD**	**Present**	1.41(1.25 to 1.60)	<0.001
**Severe NEC**	**Present**	1.43 (1.10 to 1.85)	0.007
**Brain injury**	**Present**	1.39 (1.19 to 1.62)	<0.001
**Neonatal discharge timing**	**Early (<25th centile PMA)**	1.14 (1.02 to 1.27)	0.03
	**Not early**	Reference	-
		**Predicted hazard ratio on day 1**	
**Season (Predicted hazard ratio at day 1 from time dependent effect model)***	**Spring**	0.99 (0.77 to 1.26)	
	**Summer**	Reference	
	**Autumn**	2.33 (1.83 to 2.97)	
	**Winter**	2.53 (1.65 to 3.87)	

*Hazard ratio for season shown as estimated Hazard Ratio at Day 1 due to modelling for time-dependent effect

© 2024 van Hasselt TJ et al. *JAMA Network Open.*

eTable 9. Flexible Parametric Model for Unplanned PICU Admission From Home, Including Children Discharged Home at 33 weeks' PMA or Later, Analysis for Children Born Earlier Than 24 Weeks' to 27 Weeks' Gestation and Those Born 28 to 31 Weeks' Gestation

		Subgroup: <24 to 27 weeks gestation (n=10,398)		Subgroup: 28 to 31 weeks gestation (n=29,640)	
**Variables**		**Adjusted hazard ratio (95% confidence interval)**	***p***	**Adjusted hazard ratio (95% confidence interval)**	***p***
**Gestation at birth (weeks)**	**<24**	1.71 (1.24 to 2.36)	0.001	-	-
	**24**	1.71 (1.35 to 2.16)	<0.001	-	-
	**25**	1.47 (1.18 to 1.83)	0.001	-	-
	**26**	1.35 (1.10 to 1.66)	0.004	-	-
	**27**	Reference	-	-	-
	**28**	-	-	1.25 (1.05 to 1.49)	0.01
	**29**	-	-	1.24 (1.05 to 1.46)	0.01
	**30**	-	-	1.14 (0.98 to 1.34)	0.09
	**31**	-	-	Reference	-
**Sex**	**Male**	1.18 (1.02 to 1.37)	0.03	1.31 (1.16 to 1.48)	<0.001
	**Female**	Reference	-	Reference	-
**Small for gestational age**	**Present**	1.35 (0.99 to 1.83)	0.06	1.17 (0.96 to 1.42)	0.10
**BPD**	**Present**	1.49 (1.22 to 1.81)	<0.001	1.33 (1.14 to 1.56)	<0.001
**Severe NEC**	**Present**	1.32 (0.98 to 1.79)	0.07	1.84 (1.10 to 3.07)	0.02
**Brain injury**	**Present**	1.23 (1.02 to 1.49)	0.03	1.82 (1.42 to 2.35)	<0.001
**Neonatal discharge timing**	**Early (<25th centile PMA)**	1.01 (0.84 to 1.23)	0.89	1.22 (1.06 to 1.34)	0.005
	**Not early**	Reference	-	Reference	-
		**Predicted hazard ratio on day 1**			
**Season (Predicted hazard ratio at day 1 from time dependent effect model)***	**Spring**	1.05 (0.70 to 1.56)		0.93 (0.68 to 1.29)	
	**Summer**	Reference		Reference	
	**Autumn**	1.62 (1.09 to 2.41)		2.99 (2.20 to 4.06)	
	**Winter**	1.93 (0.96 to 3.88)		3.15 (1.85 to 5.37)	

*Hazard ratio for season shown as estimated Hazard Ratio at Day 1 due to modelling for time-dependent effect

© 2024 van Hasselt TJ et al. *JAMA Network Open.*

eFigure 1. Schoenfeld Plots

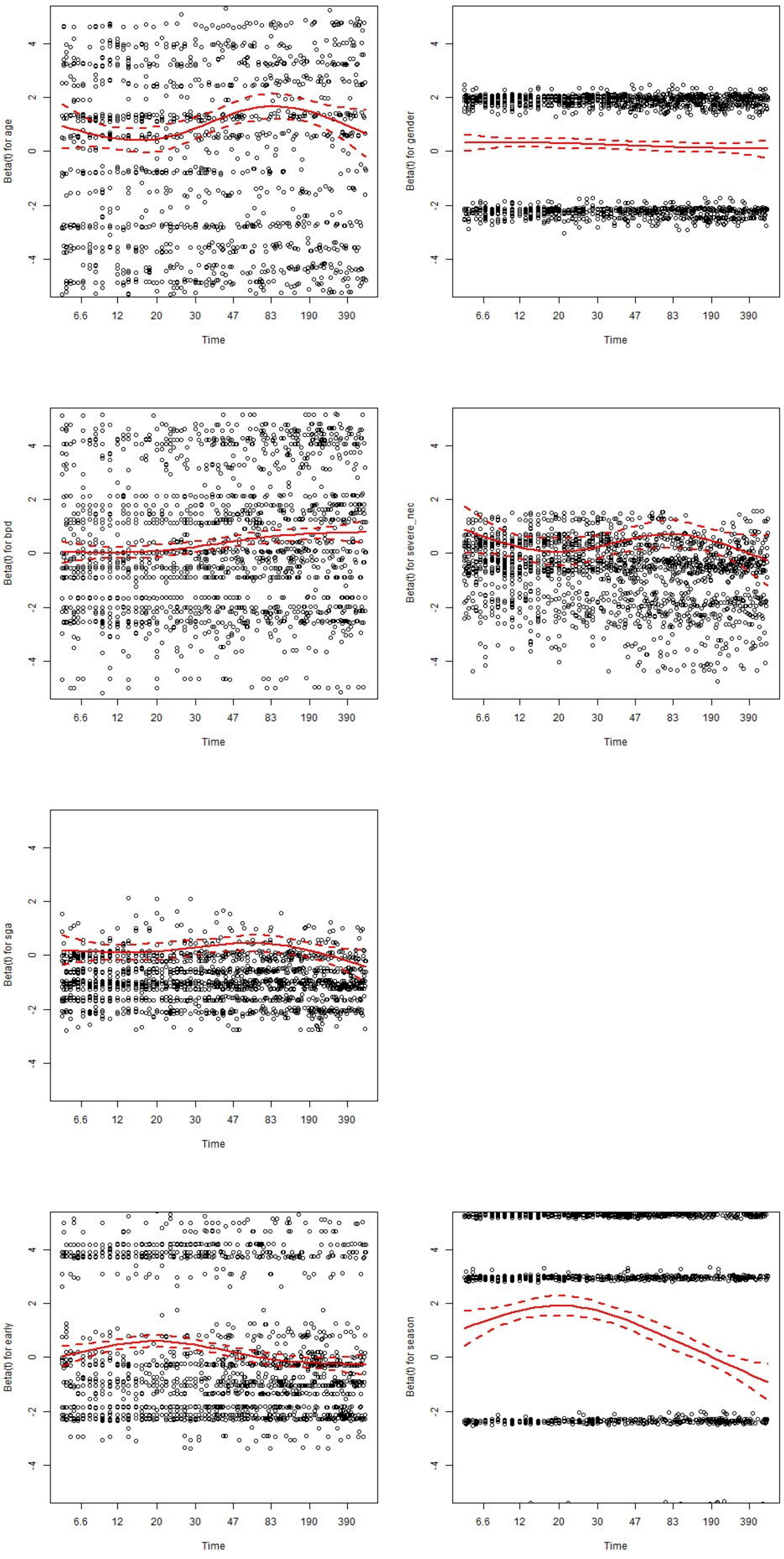

© 2024 van Hasselt TJ et al. *JAMA Network Open.*

eFigure 2. Hazard Ratio for Unplanned PICU Admission Over the First 100 Days From Neonatal Discharge for Season of Neonatal Discharge for Primary Analysis

Reference group was summer discharge

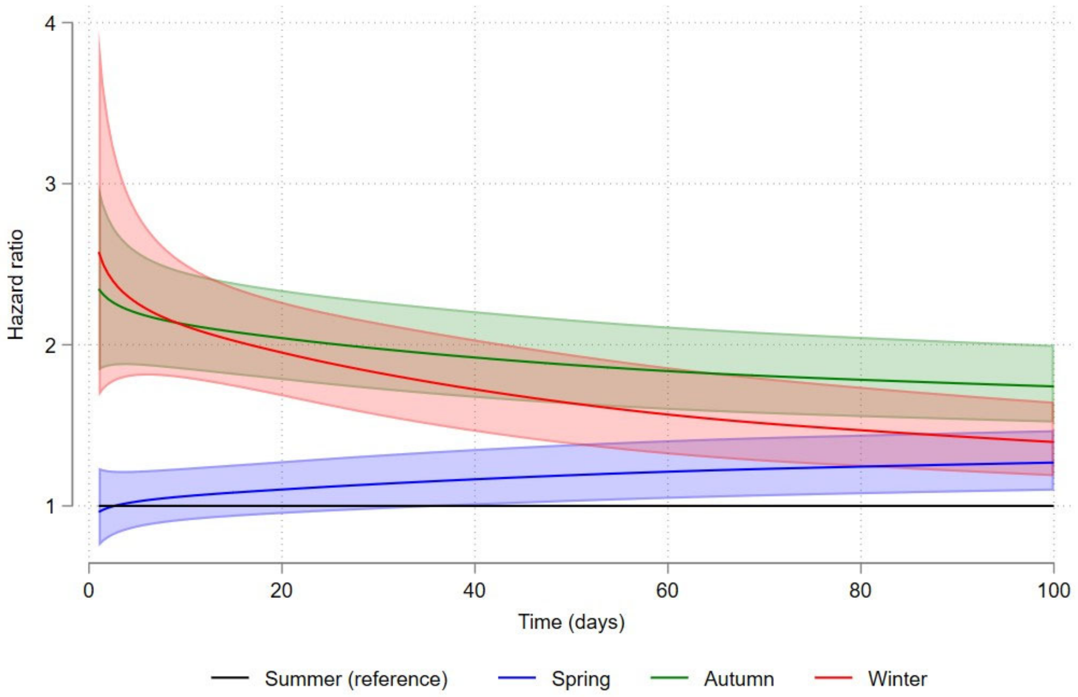

© 2024 van Hasselt TJ et al. *JAMA Network Open.*

eFigure 3. Graph of Estimated Hazard Ratio for Unplanned PICU Admission on Day 1 Following Neonatal Discharge, by Month of Neonatal Discharge

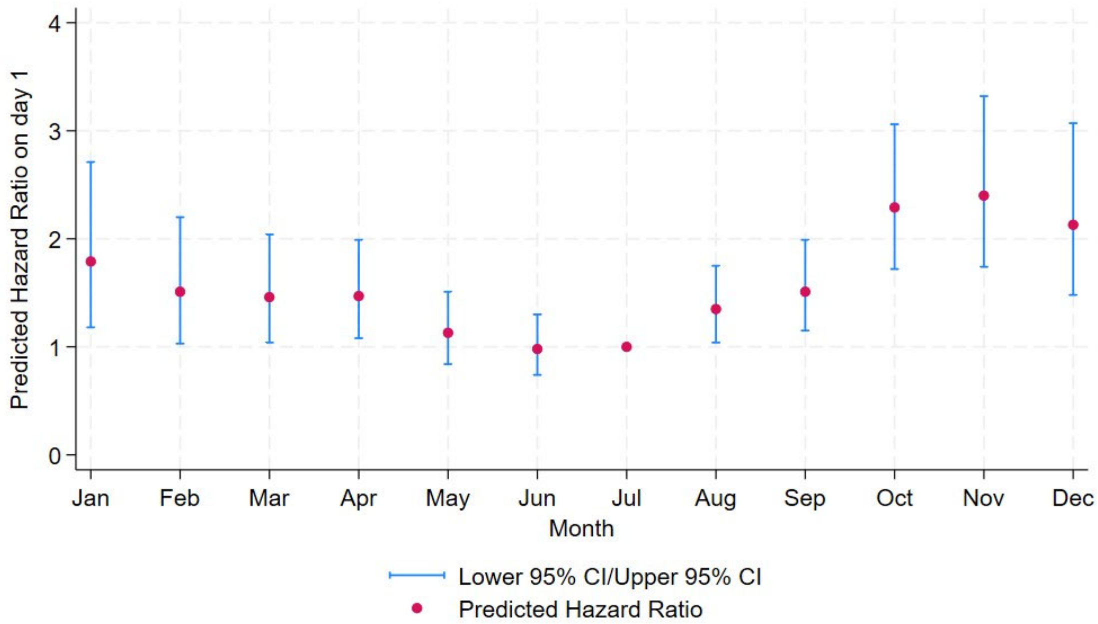

© 2024 van Hasselt TJ et al. *JAMA Network Open.*
