# Supplementary material for: Timing of Neonatal Discharge and Unplanned Readmission to PICUs Among Infants Born Preterm
Source: JAMA Netw Open. 2024 Nov 14;7(11):e2444909. doi: 10.1001/jamanetworkopen.2024.44909 (PMC11565260; doi:10.1001/jamanetworkopen.2024.44909)
Supplement: Supplement 2. — Nonauthor Collaborators [file jamanetwopen-e2444909-s002.pdf]

Supplemental Online Content: Nonauthor Collaborators
*First name, last name, and suffix (if applicable) are required and will appear in PubMed.

*Group Name(s): United Kingdom Neonatal Collaborative and the Paediatric Critical Care Society Study Group (PCCS-SG)							
***First Name and Middle Initial(s)**	***Last Name**	***Suffix (eg, Jr, III)**	Academic Degrees	Institution	Location (city, state/province, country)	Role or Contribution, eg, chair, principal investigator	Group (if more than 1 Group listed in the byline) and/or Subgroup (eg, Steering Committee)
Lyvonne	Tume		PhD	Edge Hill University	Ormskirk, England	Deputy Chair of PCCS-SG	PCCS-SG
Padmanabhan	Ramnarayan		PhD	Imperial College London	London, England	Chair of PCCS-SG	PCCS-SG
Saulius	Satas			Aberdeen Maternity Hospital,	Scotland	UKNC Lead	UK Neonatal Collaborative
Matthew	Babirecki			Airedale General Hospital	England	UKNC Lead	UK Neonatal Collaborative
Rebecca	Kettle			Alder Hey	England	UKNC Lead	UK Neonatal Collaborative
Damien	Armstrong			Altnagelvin Area Hospital	Northern Ireland	UKNC Lead	UK Neonatal Collaborative
Sanjeev	Bali			Antrim Area Hospital	Northern Ireland	UKNC Lead	UK Neonatal Collaborative
Anand	Kamalanathan			Arrowe Park Hospital	England	UKNC Lead	UK Neonatal Collaborative
Clare	Cane			Barnet Hospital	England	UKNC Lead	UK Neonatal Collaborative
Kavi	Aucharaz			Barnsley District General Hospital	England	UKNC Lead	UK Neonatal Collaborative
Rathod	Poorva			Basildon Hospital	England	UKNC Lead	UK Neonatal Collaborative
Jummy	Awoseyila			Basingstoke & North Hampshire Hospital	England	UKNC Lead	UK Neonatal Collaborative
L M	Wong			Bassetlaw District General Hospital	England	UKNC Lead	UK Neonatal Collaborative
Anita	Mittal			Bedford Hospital	England	UKNC Lead	UK Neonatal Collaborative
Penny	Broggio			Birmingham City Hospital	England	UKNC Lead	UK Neonatal Collaborative

Supplemental Online Content: Nonauthor Collaborators
*First name, last name, and suffix (if applicable) are required and will appear in PubMed.

***First Name and Middle Initial(s)**	***Last Name**	***Suffix (eg, Jr, III)**	Academic Degrees	Institution	Location (city, state/province, country)	Role or Contribution, eg, chair, principal investigator	Group (if more than 1 Group listed in the byline) and/or Subgroup (eg, Steering Committee)
Pinki	Surana			Birmingham Heartlands Hospital	England	UKNC Lead	UK Neonatal Collaborative
Matt	Nash			Birmingham Women's Hospital	England	UKNC Lead	UK Neonatal Collaborative
Clare	Irving			Borders General Hospital, Melrose	Scotland	UKNC Lead	UK Neonatal Collaborative
Sam	Sam Wallis			Bradford Royal Infirmary	England	UKNC Lead	UK Neonatal Collaborative
Ahmed	Hassan			Broomfield Hospital, Chelmsford	England	UKNC Lead	UK Neonatal Collaborative
Karin	Schwarz			Calderdale Royal Hospital	England	UKNC Lead	UK Neonatal Collaborative
Shu-Ling	Chuang			Chelsea & Westminster Hospital	England	UKNC Lead	UK Neonatal Collaborative
Penelope	Young			Chesterfield & North Derbyshire Royal Hospital	England	UKNC Lead	UK Neonatal Collaborative
Romona	Onita			Colchester General Hospital	England	UKNC Lead	UK Neonatal Collaborative
Mani	Kandasamy			Conquest Hospital	England	UKNC Lead	UK Neonatal Collaborative
Stephen	Brearey			Countess of Chester Hospital	England	UKNC Lead	UK Neonatal Collaborative
Alison	Verner			Craigavon Area Hospital and Daisy Hill Hospital	Northern Ireland	UKNC Lead	UK Neonatal Collaborative
Tim	Adams			Cross House Hospital, Kilmarnock	Scotland	UKNC Lead	UK Neonatal Collaborative
Joselyn	Morris			Croydon University Hospital	England	UKNC Lead	UK Neonatal Collaborative
Rachel	Smith			Cumberland Infirmary	England	UKNC Lead	UK Neonatal Collaborative
Bharath	Gowda			Darent Valley Hospital	England	UKNC Lead	UK Neonatal Collaborative

Supplemental Online Content: Nonauthor Collaborators
*First name, last name, and suffix (if applicable) are required and will appear in PubMed.

***First Name and Middle Initial(s)**	***Last Name**	***Suffix (eg, Jr, III)**	Academic Degrees	Institution	Location (city, state/province, country)	Role or Contribution, eg, chair, principal investigator	Group (if more than 1 Group listed in the byline) and/or Subgroup (eg, Steering Committee)
Mehdi	Garbash			Darlington Memorial Hospital	England	UKNC Lead	UK Neonatal Collaborative
Alex	Allwood			Derriford Hospital	England	UKNC Lead	UK Neonatal Collaborative
Vijaya	Hebbar			Diana Princess of Wales Hospital	England	UKNC Lead	UK Neonatal Collaborative
Nigel	Brooke			Doncaster Royal Infirmary	England	UKNC Lead	UK Neonatal Collaborative
Claire	Hollinsworth			Dorset County Hospital	England	UKNC Lead	UK Neonatal Collaborative
Andrew	Eccleston			Dumfries and Galloway Royal Infirmary	Scotland	UKNC Lead	UK Neonatal Collaborative
Toria	Klutse			East Surrey Hospital	England	UKNC Lead	UK Neonatal Collaborative
Clare	Sturdy			Epsom General Hospital	England	UKNC Lead	UK Neonatal Collaborative
Dominic	O'Reilly			Forth Valley Hospital, Larbert	Scotland	UKNC Lead	UK Neonatal Collaborative
Sathish	Krishnan			Frimley Park Hospital	England	UKNC Lead	UK Neonatal Collaborative
Maria	Hadjicosta			Furness General Hospital	England	UKNC Lead	UK Neonatal Collaborative
Sabyasachi	Chowdhury			George Eliot Hospital	England	UKNC Lead	UK Neonatal Collaborative
Ambrose	Onibere			Glan Clwyd Hospital	Wales	UKNC Lead	UK Neonatal Collaborative
Prem	Pitchaikani			Glangwili General Hospital	Wales	UKNC Lead	UK Neonatal Collaborative
Shyam	Bhakthavalsala			Gloucester Royal Hospital	England	UKNC Lead	UK Neonatal Collaborative
Daniel	Dogar			Good Hope Hospital	England	UKNC Lead	UK Neonatal Collaborative

Supplemental Online Content: Nonauthor Collaborators
*First name, last name, and suffix (if applicable) are required and will appear in PubMed.

***First Name and Middle Initial(s)**	***Last Name**	***Suffix (eg, Jr, III)**	Academic Degrees	Institution	Location (city, state/province, country)	Role or Contribution, eg, chair, principal investigator	Group (if more than 1 Group listed in the byline) and/or Subgroup (eg, Steering Committee)
Girish	Gowda			Great Western Hospital	England	UKNC Lead	UK Neonatal Collaborative
Karen	Turnock			Guy's & St Thomas' Hospital	England	UKNC Lead	UK Neonatal Collaborative
Patricia	Gilbertson			Harrogate District Hospital	England	UKNC Lead	UK Neonatal Collaborative
Cath	Seagrave			Hereford County Hospital	England	UKNC Lead	UK Neonatal Collaborative
Tristan	Bate			Hillingdon Hospital	England	UKNC Lead	UK Neonatal Collaborative
Hilary	Dixon			Hinchingbrooke Hospital	England	UKNC Lead	UK Neonatal Collaborative
Narendra	Aladangady			Homerton Hospital	England	UKNC Lead	UK Neonatal Collaborative
Hassan	Gaili			Hull Royal infirmary	England	UKNC Lead	UK Neonatal Collaborative
Matthew	James			Ipswich Hospital	England	UKNC Lead	UK Neonatal Collaborative
M	Lal			James Cook University Hospital	England	UKNC Lead	UK Neonatal Collaborative
Oluseun	Tayo			James Paget Hospital	England	UKNC Lead	UK Neonatal Collaborative
Abraham	Isaac			Kettering General Hospital	England	UKNC Lead	UK Neonatal Collaborative
Carolina	Zorro			Kings College Hospital	England	UKNC Lead	UK Neonatal Collaborative
Dhaval	Dave			King's Mill Hospital	England	UKNC Lead	UK Neonatal Collaborative
Jonathan	Filkin			Kingston Hospital	England	UKNC Lead	UK Neonatal Collaborative

Supplemental Online Content: Nonauthor Collaborators
*First name, last name, and suffix (if applicable) are required and will appear in PubMed.

***First Name and Middle Initial(s)**	***Last Name**	***Suffix (eg, Jr, III)**	Academic Degrees	Institution	Location (city, state/province, country)	Role or Contribution, eg, chair, principal investigator	Group (if more than 1 Group listed in the byline) and/or Subgroup (eg, Steering Committee)
Savi	Sivashankar			Lancashire Women and Newborn Centre	England	UKNC Lead	UK Neonatal Collaborative
Hannah	Shore			Leeds General Infirmary and St James University Hospital	England	UKNC Lead	UK Neonatal Collaborative
Jo	Behrsin			Leicester General Hospital and Leicester Royal Infirmary	England	UKNC Lead	UK Neonatal Collaborative
Michael	Grosdenier			Leighton Hospital	England	UKNC Lead	UK Neonatal Collaborative
Ruchika	Gupta			Lincoln County Hospital and Pilgrim Hospital	England	UKNC Lead	UK Neonatal Collaborative
Ather	Ahmed			Lister Hospital	England	UKNC Lead	UK Neonatal Collaborative
Nim	Subhedar			Liverpool Women's Hospital	England	UKNC Lead	UK Neonatal Collaborative
Jennifer	Birch			Luton & Dunstable Hospital	England	UKNC Lead	UK Neonatal Collaborative
Surendran	Chandrasekaran			Macclesfield District General Hospital	England	UKNC Lead	UK Neonatal Collaborative
Ashok	Karupaiah			Manor Hospital (Walsall)	England	UKNC Lead	UK Neonatal Collaborative
Ghada	Ramadan			Medway Maritime Hospital	England	UKNC Lead	UK Neonatal Collaborative
I	Misra			Milton Keynes General Hospital	England	UKNC Lead	UK Neonatal Collaborative
Chris	Knight			Musgrove Park Hospital	England	UKNC Lead	UK Neonatal Collaborative
Richard	Heaver			New Cross Hospital	England	UKNC Lead	UK Neonatal Collaborative
Mohammad	Alam			Newham General Hospital	England	UKNC Lead	UK Neonatal Collaborative
Bhushan	Bhushan			Ninewells Hospital, Dundee	Scotland	UKNC Lead	UK Neonatal Collaborative

Supplemental Online Content: Nonauthor Collaborators
*First name, last name, and suffix (if applicable) are required and will appear in PubMed.

***First Name and Middle Initial(s)**	***Last Name**	***Suffix (eg, Jr, III)**	Academic Degrees	Institution	Location (city, state/province, country)	Role or Contribution, eg, chair, principal investigator	Group (if more than 1 Group listed in the byline) and/or Subgroup (eg, Steering Committee)
Prakash	Thiagarajan			Nobles Hospital	England	UKNC Lead	UK Neonatal Collaborative
Priya	Muthukumar			Norfolk & Norwich University Hospital	England	UKNC Lead	UK Neonatal Collaborative
Tiziana	Fragapane			North Devon District Hospital	England	UKNC Lead	UK Neonatal Collaborative
Bivan	Saha			North Manchester General Hospital	England	UKNC Lead	UK Neonatal Collaborative
Cheentan	Singh			North Middlesex University Hospital	England	UKNC Lead	UK Neonatal Collaborative
Nick	Barnes			Northampton General Hospital	England	UKNC Lead	UK Neonatal Collaborative
Sangeeta	Tiwary			Northumbria Specialist Emergency Care Hospital	England	UKNC Lead	UK Neonatal Collaborative
Richard	Nicholl			Northwick Park Hospital	England	UKNC Lead	UK Neonatal Collaborative
Dush	Batra			Nottingham City Hospital and Nottingham University Hospital (QMC)	England	UKNC Lead	UK Neonatal Collaborative
Victoria	Nesbitt			Ormskirk District General Hospital	England	UKNC Lead	UK Neonatal Collaborative
Amit	Gupta			Oxford University Hospitals, John Radcliffe Hospital	England	UKNC Lead	UK Neonatal Collaborative
Katharine	McDevitt			Peterborough City Hospital	England	UKNC Lead	UK Neonatal Collaborative
David	Gibson			Pinderfields General Hospital	England	UKNC Lead	UK Neonatal Collaborative
Peter	Mcewan			Poole General Hospital	England	UKNC Lead	UK Neonatal Collaborative
David	Deekollu			Prince Charles Hospital	Wales	UKNC Lead	UK Neonatal Collaborative

*First name, last name, and suffix (if applicable) are required and will appear in PubMed.

*First Name and Middle Initial(s)	*Last Name	*Suffix (eg, Jr, III)	Academic Degrees	Institution	Location (city, state/province, country)	Role or Contribution, eg, chair, principal investigator	Group (if more than 1 Group listed in the byline) and/or Subgroup (eg, Steering Committee)
Sanath	Reddy			Princess Alexandra Hospital	England	UKNC Lead	UK Neonatal Collaborative
Mark	Johnson			Princess Anne Hospital	England	UKNC Lead	UK Neonatal Collaborative
Abby	Parish			Princess of Wales Hospital	Wales	UKNC Lead	UK Neonatal Collaborative
Cassie	Lawn			Princess Royal Hospital and Royal Sussex County Hospital	England	UKNC Lead	UK Neonatal Collaborative
Patricia	Cowley			Princess Royal Hospital Telford	England	UKNC Lead	UK Neonatal Collaborative
Carolyn	Abernethy			Princess Royal Maternity Hospital, Glasgow	Scotland	UKNC Lead	UK Neonatal Collaborative
Rashmi	Gandhi			Princess Royal University Hospital	England	UKNC Lead	UK Neonatal Collaborative
Charlotte	Groves			Queen Alexandra Hospital	England	UKNC Lead	UK Neonatal Collaborative
Lidia	Tyszczuk			Queen Charlotte's Hospital	England	UKNC Lead	UK Neonatal Collaborative
Shilpa	Ramesh			Queen Elizabeth Hospital, Gateshead	England	UKNC Lead	UK Neonatal Collaborative
Salamatu	Jalloh			Queen Elizabeth Hospital, King's Lynn	England	UKNC Lead	UK Neonatal Collaborative
Julia	Croft			Queen Elizabeth Hospital, Woolwich	England	UKNC Lead	UK Neonatal Collaborative
Bushra	Abdul-Malik			Queen Elizabeth the Queen Mother Hospital	England	UKNC Lead	UK Neonatal Collaborative
Dominic	Muogbo			Queen's Hospital, Burton on Trent	England	UKNC Lead	UK Neonatal Collaborative
Ambalika	Das			Queen's Hospital, Romford	England	UKNC Lead	UK Neonatal Collaborative
Khalid	Mannan			Queen's Hospital, Romford 2	England	UKNC Lead	UK Neonatal Collaborative

Supplemental Online Content: Nonauthor Collaborators
*First name, last name, and suffix (if applicable) are required and will appear in PubMed.

***First Name and Middle Initial(s)**	***Last Name**	***Suffix (eg, Jr, III)**	Academic Degrees	Institution	Location (city, state/province, country)	Role or Contribution, eg, chair, principal investigator	Group (if more than 1 Group listed in the byline) and/or Subgroup (eg, Steering Committee)
P	Van Der Heide			Raigmore Hospital, Inverness	Scotland	UKNC Lead	UK Neonatal Collaborative
Rajiv	Chaudhary			Rosie Maternity Hospital, Addenbrookes	England	UKNC Lead	UK Neonatal Collaborative
Soma	Sengupta			Rotherham District General Hospital	England	UKNC Lead	UK Neonatal Collaborative
Christos	Zipitis			Royal Albert Edward Infirmary	England	UKNC Lead	UK Neonatal Collaborative
Hilary	Conetta			Royal Alexandra Hospital, Paisley	Scotland	UKNC Lead	UK Neonatal Collaborative
Kemy	Naidoo			Royal Berkshire Hospital	England	UKNC Lead	UK Neonatal Collaborative
Dinakar	Seshadri			Royal Bolton Hospital	England	UKNC Lead	UK Neonatal Collaborative
Chris	Warren			Royal Cornwall Hospital	England	UKNC Lead	UK Neonatal Collaborative
Nigel	Ruggins			Royal Derby Hospital	England	UKNC Lead	UK Neonatal Collaborative
Chrissie	Oliver			Royal Devon & Exeter Hospital	England	UKNC Lead	UK Neonatal Collaborative
Lucinda	Winckworth			Royal Hampshire County Hospital	England	UKNC Lead	UK Neonatal Collaborative
Joanne	Fedee			Royal Lancaster Infirmary	England	UKNC Lead	UK Neonatal Collaborative
Stan	Craig			Royal Maternity Hospital	Northern Ireland	UKNC Lead	UK Neonatal Collaborative
Anitha	Vayalakkad			Royal Oldham Hospital	England	UKNC Lead	UK Neonatal Collaborative
Richa	Gupta			Royal Preston Hospital	England	UKNC Lead	UK Neonatal Collaborative
Julia	Uffindell			Royal Stoke University Hospital	England	UKNC Lead	UK Neonatal Collaborative

Supplemental Online Content: Nonauthor Collaborators
*First name, last name, and suffix (if applicable) are required and will appear in PubMed.

***First Name and Middle Initial(s)**	***Last Name**	***Suffix (eg, Jr, III)**	Academic Degrees	Institution	Location (city, state/province, country)	Role or Contribution, eg, chair, principal investigator	Group (if more than 1 Group listed in the byline) and/or Subgroup (eg, Steering Committee)
Jo	MacLeod			Royal Surrey County Hospital	England	UKNC Lead	UK Neonatal Collaborative
Rebecca	Winterson			Royal United Hospital	England	UKNC Lead	UK Neonatal Collaborative
Naveen	Athiraman			Royal Victoria Infirmary	England	UKNC Lead	UK Neonatal Collaborative
Muhammad	Khurshid			Russells Hall Hospital	England	UKNC Lead	UK Neonatal Collaborative
Jim	Baird			Salisbury District Hospital	England	UKNC Lead	UK Neonatal Collaborative
Adedayo	Owoeye			Scarborough General Hospital	England	UKNC Lead	UK Neonatal Collaborative
Umapathee	Majuran			Scunthorpe General Hospital	England	UKNC Lead	UK Neonatal Collaborative
Richard	Lindley			Sheffield Children's Hospital	England	UKNC Lead	UK Neonatal Collaborative
Ben	Stenson			Simpsons Centre for Reproductive Health, Royal Infirmary of Edinburgh	Scotland	UKNC Lead	UK Neonatal Collaborative
Arun	Ramachandran			Singleton Hospital	Wales	UKNC Lead	UK Neonatal Collaborative
Damien	Armstrong			South West Acute Hospital (SWAH)	Northern Ireland	UKNC Lead	UK Neonatal Collaborative
Vineet	Vineet Gupta			Southend Hospital	England	UKNC Lead	UK Neonatal Collaborative
Faith	Emery			Southmead Hospital	England	UKNC Lead	UK Neonatal Collaborative
Madhavi	Parvathareddy			Southmead Hospital	England	UKNC Lead	UK Neonatal Collaborative
Donovan	Duffy			St George's Hospital	England	UKNC Lead	UK Neonatal Collaborative

Supplemental Online Content: Nonauthor Collaborators
*First name, last name, and suffix (if applicable) are required and will appear in PubMed.

***First Name and Middle Initial(s)**	***Last Name**	***Suffix (eg, Jr, III)**	Academic Degrees	Institution	Location (city, state/province, country)	Role or Contribution, eg, chair, principal investigator	Group (if more than 1 Group listed in the byline) and/or Subgroup (eg, Steering Committee)
Salim	Yasin			St Helier Hospital	England	UKNC Lead	UK Neonatal Collaborative
Helen	Rhodes			St John’s Hospital, Livingston	Scotland	UKNC Lead	UK Neonatal Collaborative
Akinsola	Ogundiya			St Mary's Hospital, IOW	England	UKNC Lead	UK Neonatal Collaborative
Lidia	Lidia			St Mary's Hospital, London	England	UKNC Lead	UK Neonatal Collaborative
Arin	Mukherjee			St Mary's Hospital, Manchester	England	UKNC Lead	UK Neonatal Collaborative
Pamela	Cairns			St Michael's Hospital	England	UKNC Lead	UK Neonatal Collaborative
Vennila	Ponnusamy			St Peter's Hospital	England	UKNC Lead	UK Neonatal Collaborative
Victoria	Sharp			St Richard's Hospital	England	UKNC Lead	UK Neonatal Collaborative
Carrie	Heal			Stepping Hill Hospital	England	UKNC Lead	UK Neonatal Collaborative
Sanjay	Salgia			Stoke Mandeville Hospital	England	UKNC Lead	UK Neonatal Collaborative
Imran	Ahmed			Sunderland Royal Hospital	England	UKNC Lead	UK Neonatal Collaborative
Helen	Purves			Tameside General Hospital	England	UKNC Lead	UK Neonatal Collaborative
Anitha	James			The Grange University Hospital	Wales	UKNC Lead	UK Neonatal Collaborative
Porus	Bastani			The Jessop Wing, Sheffield	England	UKNC Lead	UK Neonatal Collaborative
AM	Heuchan			The Queen Elizabeth University Hospital Glasgow	Scotland	UKNC Lead	UK Neonatal Collaborative
Eleanor	Bond			The Royal Free Hospital	England	UKNC Lead	UK Neonatal Collaborative

Supplemental Online Content: Nonauthor Collaborators
*First name, last name, and suffix (if applicable) are required and will appear in PubMed.

***First Name and Middle Initial(s)**	***Last Name**	***Suffix (eg, Jr, III)**	Academic Degrees	Institution	Location (city, state/province, country)	Role or Contribution, eg, chair, principal investigator	Group (if more than 1 Group listed in the byline) and/or Subgroup (eg, Steering Committee)
Divyen	Shah			The Royal London Hospital - Constance Green	England	UKNC Lead	UK Neonatal Collaborative
Esther	Morris			Torbay Hospital	England	UKNC Lead	UK Neonatal Collaborative
Mithun	Urs			Tunbridge Wells Hospital	England	UKNC Lead	UK Neonatal Collaborative
Nita	Saxena			Ulster Hospital	Northern Ireland	UKNC Lead	UK Neonatal Collaborative
Giles	Kendall			University College Hospital	England	UKNC Lead	UK Neonatal Collaborative
Puneet	Nath			University Hospital Coventry	England	UKNC Lead	UK Neonatal Collaborative
Igor	Fierens			University Hospital Lewisham	England	UKNC Lead	UK Neonatal Collaborative
Mehdi	Garbash			University Hospital of North Durham	England	UKNC Lead	UK Neonatal Collaborative
Hari	Kumar			University Hospital of North Tees	England	UKNC Lead	UK Neonatal Collaborative
Nitin	Goel			University Hospital of Wales	Wales	UKNC Lead	UK Neonatal Collaborative
Peter	Curtis			Victoria Hospital, Blackpool	England	UKNC Lead	UK Neonatal Collaborative
Laura	Stewart			Victoria Hospital, Kirkcaldy	Scotland	UKNC Lead	UK Neonatal Collaborative
Delyth	Webb			Warrington Hospital	England	UKNC Lead	UK Neonatal Collaborative
Sumedha	Bird			Warwick Hospital	England	UKNC Lead	UK Neonatal Collaborative
Sankara	Narayanan			Watford General Hospital	England	UKNC Lead	UK Neonatal Collaborative
Yee Mon	Aung			West Cumberland Hospital	England	UKNC Lead	UK Neonatal Collaborative

Supplemental Online Content: Nonauthor Collaborators
*First name, last name, and suffix (if applicable) are required and will appear in PubMed.

***First Name and Middle Initial(s)**	***Last Name**	***Suffix (eg, Jr, III)**	Academic Degrees	Institution	Location (city, state/province, country)	Role or Contribution, eg, chair, principal investigator	Group (if more than 1 Group listed in the byline) and/or Subgroup (eg, Steering Committee)
Elizabeth	Eyre			West Middlesex University Hospital	England	UKNC Lead	UK Neonatal Collaborative
Tayyaba	Aamir			West Suffolk Hospital	England	UKNC Lead	UK Neonatal Collaborative
Angela	Yannoulias			Wexham Park Hospital	England	UKNC Lead	UK Neonatal Collaborative
Caroline	Sullivan			Whipps Cross University Hospital	England	UKNC Lead	UK Neonatal Collaborative
Ros	Garr			Whiston Hospital	England	UKNC Lead	UK Neonatal Collaborative
Wynne	Leith			Whittington Hospital	England	UKNC Lead	UK Neonatal Collaborative
Shaveta	Mulla			William Harvey Hospital	England	UKNC Lead	UK Neonatal Collaborative
Lorraine	McGlory			Wishaw General Hospital	Scotland	UKNC Lead	UK Neonatal Collaborative
Anna	Gregory			Worcestershire Royal Hospital	England	UKNC Lead	UK Neonatal Collaborative
Edward	Yates			Worthing Hospital	England	UKNC Lead	UK Neonatal Collaborative
Artur	Abelian			Wrexham Maelor Hospital	Wales	UKNC Lead	UK Neonatal Collaborative
Abijeet	Godhamgaonkar			Wythenshawe Hospital	England	UKNC Lead	UK Neonatal Collaborative
Siba	Paul			Yeovil District Hospital	England	UKNC Lead	UK Neonatal Collaborative
Sundeep	Sandhu			York District Hospital	England	UKNC Lead	UK Neonatal Collaborative
Shakir	Saeed			Ysbyty Gwynedd	Wales	UKNC Lead	UK Neonatal Collaborative
Nicola	Mackintosh		PhD	s	Leicester, UK	Advisory Panel Member	

Supplemental Online Content: Nonauthor Collaborators
*First name, last name, and suffix (if applicable) are required and will appear in PubMed.

***First Name and Middle Initial(s)**	***Last Name**	***Suffix (eg, Jr, III)**	Academic Degrees	Institution	Location (city, state/province, country)	Role or Contribution, eg, chair, principal investigator	Group (if more than 1 Group listed in the byline) and/or Subgroup (eg, Steering Committee)
Jonathan	Cusack		MBChB	Leicester Neonatal Service	Leicester, UK	Advisory Panel Member	
Jennifer J	Kurinczuk		PhD	National Perinatal Epidemiology Unit (NPEU), University of Oxford	Oxford, UK	Advisory Panel Member	
Patrick	Davies		BMBS	Nottingham University Hospitals NHS Trust	Nottingham, UK	Advisory Panel Member	
Joseph	Manning		PhD	School of Healthcare, University of Leicester, and Nottingham Children's Hospital	Leicester, UK	Advisory Panel Member	
